# Endovascular Treatment for Femoropopliteal Arterial Lesions with a Focus on Long-Term Limb Prognosis

**DOI:** 10.3400/avd.ra.26-00050

**Published:** 2026-05-12

**Authors:** Shigeo Ichihashi

**Affiliations:** Department of Diagnostic and Interventional Radiology, Nara Medical University, Kashihara, Nara, Japan

**Keywords:** lower extremity artery disease, endovascular treatment, chronic limb threatening ischemia

## Abstract

Femoropopliteal (FP) arterial lesions represent the most prevalent and challenging targets for endovascular therapy (EVT) in patients with lower extremity artery disease (LEAD). Despite significant advances in device technology, FP interventions continue to be limited by high rates of restenosis and occlusion. To date, no conclusive evidence has demonstrated the superiority of any specific endovascular device strategy for FP artery disease. Importantly, stent restenosis is characterized by longer lesion lengths and a higher incidence of occlusive failure, which may substantially compromise the feasibility of subsequent reintervention.

## Introduction

Lower extremity artery disease (LEAD) represents a systemic manifestation of atherosclerosis and is associated with substantial morbidity and mortality. Among patients with LEAD, femoropopliteal (FP) arterial lesions constitute the most common and clinically challenging targets for endovascular treatment (EVT). This complexity arises from the frequent presence of longer lesion lengths, chronic total occlusions, severe calcification, and pronounced biomechanical stress caused by knee flexion and limb movement, all of which adversely affect procedural durability and long-term outcomes.^1)^

Over the past 2 decades, EVT has become the first-line revascularization strategy for FP disease. Technological advances, including drug-coated balloons (DCBs), drug-eluting stents (DESs), and stent grafts, have substantially improved short- and mid-term patency rates. Nevertheless, FP interventions continue to be characterized by a relatively high incidence of restenosis and occlusion. Importantly, emerging evidence suggests that device-specific patterns of loss of patency, rather than patency itself, may be more relevant to long-term limb outcomes.

Accordingly, this review summarizes contemporary clinical outcomes of FP interventions using currently available endovascular devices, with a particular emphasis on device-specific failure modes.

## Emergence of Paclitaxel Devices Improves Outcomes

Paclitaxel, an antiproliferative agent originally developed for cancer therapy, has been incorporated into angioplasty balloons and stents to suppress neointimal hyperplasia and improve outcomes after FP endovascular intervention. The clinical benefit of paclitaxel-eluting stents was first established by the Zilver PTX (Cook Medical, Bloomington, IN, USA) randomized controlled trial (RCT), which demonstrated the superiority of paclitaxel-eluting stents over balloon angioplasty and provisional bare metal stent (BMS) implantation in FP disease.^2)^ At 5 years, the DES achieved significantly higher primary patency (66.4% vs. 43.4%) and freedom from target lesion revascularization (TLR) (83.1% vs. 67.6%) compared with standard care. Importantly, clinical benefit, defined as freedom from persistent or worsening ischemic symptoms, was also superior in the DES group (79.8% vs. 59.3%).^3)^ Subsequent technological refinements led to polymer-based paclitaxel-eluting stents designed for sustained drug release. In the IMPERIAL RCT, the Eluvia DES (Boston Scientific, Marlborough, MA, USA) demonstrated superior 12-month primary patency compared with the Zilver PTX stent (86.8% vs. 81.5%).^4)^ Clinically driven TLR was numerically lower (4.5% vs. 9.0%), and stent thrombosis occurred less frequently at 12 months (1.7% vs. 4.0%). However, real-world data have revealed device-specific failure patterns that are not fully captured by the RCTs. In the CAPSICUM registry, which evaluated Eluvia DES use in more complex lesions (mean lesion length, 18.6 cm; chronic total occlusion [CTO], 53.2%; bilateral wall calcification, 41.9%), 12-month rates of restenosis and TLR were 12.9% and 6.2%, respectively.^5)^ Further analysis of restenosis revealed that approximately 71% of restenotic lesions manifested as occlusions, and 25.9% were associated with stent thrombosis.

In parallel, DCB angioplasty has emerged as an effective “leave nothing behind” strategy and has consistently demonstrated superiority over plain old balloon angioplasty (POBA). In the IN.PACT SFA (Medtronic, Dublin, Ireland), randomized trial (mean lesion length, 8.9 cm; CTO, 26%), 12-month primary patency was significantly higher with the high-dose paclitaxel DCB than with POBA (82.2% vs. 52.4%), accompanied by a marked reduction in clinically driven TLR (2.4% vs. 20.6%).^6)^ Similarly, the LEVANT 2 trial evaluated the Lutonix DCB (BD, Franklin Lakes, NJ, USA) in shorter FP lesions (mean length, 6.3 ± 4.1 cm) and reported superior primary patency at 12 months (65.2% vs. 52.6%) and comparable freedom from TLR (83.9% vs. 79.0%) compared with POBA.^7)^ The RANGER II SFA trial, which assessed a low-dose paclitaxel Ranger DCB (Boston Scientific), demonstrated higher 12-month primary patency than POBA in lesions with a mean length of 8.2 cm (82.9% vs. 66.3%).^8)^ Collectively, these pivotal trials established DCB angioplasty as a robust strategy that improves vessel patency. A key unresolved issue has been whether the paclitaxel dose of the DCBs determines clinical outcomes. The multicenter COMPARE RCT directly compared a low-dose (2.0 µg/mm^2^) Ranger DCB with a high-dose (3.5 µg/mm^2^) IN.PACT DCB for FP lesions. At 12 months, Kaplan–Meier estimates of primary patency were nearly identical between the low- and high-dose DCB groups (81.5% vs. 83.0%), with no significant differences in all-cause mortality or clinically driven TLR.^9)^ These findings were further corroborated by the PROSPECT MONSTER study, a prospective multicenter nonrandomized registry enrolling 581 Japanese patients treated either with low- or high-dose DCB. After propensity score matching, 1-year primary patency rates were comparable between groups (87.0% vs. 81.3%), supporting the concept that paclitaxel dose alone does not dictate clinical effectiveness.^10)^

To evaluate the real-world performance of DCBs, the POPCORN registry prospectively evaluated 3165 de novo or restenotic FP lesions treated with DCB angioplasty (mean lesion length, 13.5 ± 9.3 cm; CTO, 25.9%). Bailout stenting was required in only 3.5% of cases, and postprocedural slow-flow phenomena were observed in 3.9%. During a median follow-up of 14.2 months, the 12-month freedom from restenosis was 84.5%, and freedom from TLR was 91.5%.^11)^ Restenosis patterns were heterogeneous, with occlusive restenosis accounting for approximately one-third of cases. Independent predictors of restenosis included prior revascularization, smaller distal reference vessel diameter, severe calcification, CTO, low-dose DCB use, and residual stenosis.

## Comparison between DES and DCB in FP Disease

Direct comparisons between DES and DCB in FP artery disease remain limited. The REAL PTX RCT compared the Zilver PTX DES with the IN.PACT DCB in symptomatic FP lesions.^12)^ In this multicenter study, 150 patients were randomized to primary DES implantation or DCB angioplasty with provisional bailout stenting after stratification by lesion length. More than half of the lesions were CTOs, with similar mean lesion lengths (15.5 vs. 14.9 cm). Bailout stenting was required in 25.3% of the DCB arm. Primary patency at 12 months was comparable (79% vs. 80%; p = 0.96), but declined to 54% versus 38% at 36 months (p = 0.17), showing a numerical trend favoring DES during longer follow-up. The DRASTICO trial, a single-center RCT, also compared IN.PACT DCB with Zilver PTX DES in a high-risk population.^13)^ Diabetes and chronic limb-threatening ischemia were present in more than half of the patients in both arms. The mean lesion length was approximately 14 cm, and the rate of CTO approached 60%. At 12 months, target lesion restenosis (22% vs. 21%) and clinically driven TLR (14% vs. 17%) were similar, indicating that DCB was not superior to DES in complex FP lesions. The BEST-SFA trial, an investigator-initiated, prospective, multicenter RCT, enrolled 120 patients with complex FP lesions with a mean lesion length of 187.7 ± 78.3 mm and a CTO rate of 79.2%. The patients were randomized to a stent-avoiding strategy (DCB) versus a stent-preferred strategy (Eluvia DES).^14)^ At 12 months, primary patency was identical (78.2% vs. 78.6%), and freedom from major adverse events was similarly high (>90%), with events attributable to clinically driven TLR.

## Stent Grafts for Long FP Lesions

Covered stents play an important role in improving primary patency in long and complex FP arterial lesions, particularly those with CTO or in-stent restenosis (ISR). In de novo long FP lesions, the VIASTAR RCT demonstrated the superiority of heparin-bonded Viabahn stent grafts (W. L. Gore & Associates, Flagstaff, AZ, USA) over BMSs. Among lesions with a mean length of 19.0 ± 6.3 cm, 12-month primary patency was significantly higher in the stent graft group than in the BMS group (70.9% vs. 55.1%), demonstrating the efficacy of covered stents for long occlusive disease.^15)^ In the Japanese multicenter prospective VANQUISH study, clinical outcomes were evaluated in a subgroup of 343 limbs treated with full lesion coverage using Viabahn stent grafts.^16)^ The 12-month primary patency was 80.3%. However, during the first year of follow-up, 46 TLRs and 21 acute thrombotic occlusions occurred. A smaller reference vessel diameter was independently associated with loss of patency, underscoring the importance of careful vessel sizing when selecting covered stent therapy.^16)^ Covered stents have also been evaluated for the treatment of FP ISR. The RELINE RCT directly compared Viabahn stent graft implantation with balloon angioplasty for FP ISR.^17)^ At 12 months, primary patency was significantly higher in the stent graft group than in the balloon angioplasty group (74.8% vs. 28.0%), with a markedly lower rate of clinically driven TLR (27.8% vs. 80.0%).

Despite favorable patency outcomes, thrombosis represents a distinct and clinically important failure mode of covered stents. The multicenter CUCUMBER study specifically investigated the clinical consequences of Viabahn stent graft thrombosis.^18)^ Thrombosis occurred in 159 of 1215 treated patients (13%) at a median of 6.4 months after implantation, and 21 patients (13%) presented with acute limb ischemia. Among patients undergoing reintervention, including surgical bypass, endovascular reintervention, or thrombectomy, clinical outcomes were suboptimal, with a 12-month primary patency of 54.9% and freedom from major adverse limb events of 73.6%. These findings highlight that, although covered stents provide excellent initial patency in selected FP lesions, stent graft occlusion is associated with poor outcomes and may pose a substantial threat to long-term limb prognosis.

## Treatment Strategy Considering Long-Term Patient Outcomes

**Fig. 1:**
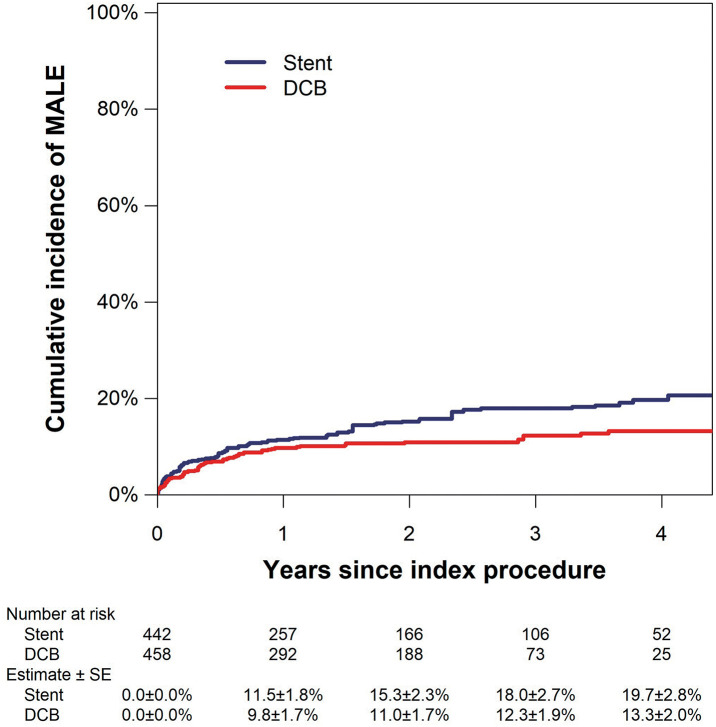
Cumulative incidence estimates of MALE in patients with CLTI after SFA EVT with a DCB or stenting after inverse probability of treatment weighting. Reprinted from Kobayashi T et al., J Vasc Surg 2025; 82: 164-72.E2,^20)^ with permission from Elsevier. MALE: major adverse limb events; CLTI: chronic limb-threatening ischemia; SFA: superficial femoral artery; EVT: endovascular therapy; DCB: drug-coated balloon

DES, DCB, and covered stents each demonstrate favorable patency under specific anatomical and clinical conditions, yet they differ substantially in their modes of failure and clinical consequences. The choice between DCBs and scaffolds has become a major interest in FP intervention, with failure modes taken into consideration. To address this clinically relevant question, a multicenter retrospective cohort study was performed to compare DES and DCB using propensity score matching.^19)^ A total of 617 FP lesions were analyzed, from which 290 DES-treated and 145 DCB-treated cases were matched. Although primary patency at 1 and 2 years was modestly but significantly higher in the DES group (84.8% and 71.1%) compared with the DCB group (81.3% and 66.6%), freedom from TLR did not differ significantly between the 2 modalities. Importantly, the mode of failure differed substantially. At the time of loss of patency, DES-treated lesions were significantly more likely to present with occlusive restenosis, longer occlusion lengths, and exacerbation of clinical symptoms compared with DCB-treated lesions. A recent multicenter observational study of 900 CLTI patients undergoing SFA intervention also compared DCB and stent placement.^20)^ The study demonstrated that, compared with stenting, DCB use was associated with a significantly lower risk of MALE (**Fig. 1**) and a lower incidence of acute occlusion. These results suggest that DCB may be a safer primary treatment option for FP lesions, given the failure modes associated with stents.

## Conclusions

To date, no definitive evidence has established the superiority of any single endovascular device strategy for FP artery disease, particularly in the setting of long, complex lesions. Device selection for FP interventions should not rely solely on primary patency or freedom from TLR at 1–2 years. Instead, greater emphasis should be placed on the anticipated mode of failure, the feasibility of reintervention or conversion to surgical bypass, and the potential impact on long-term limb outcomes.
